# Names

**Published:** 1884-01

**Authors:** Ausburn Towner


### NAMES.

AUSBURN TOWNER.

There is something in the study or observation of names that is pleasing to the least curious or most thoughtless. The old habit, attributed in a contemptuous spirit, as showing his uncultured taste to the countryman, of gazing at the signs along the business streets of a strange place, is a habit that belongs to human nature at large, as if one would judge of the persons among whom he is thrown, by the names they bear. Any peculiarity that is common among us is not noticeable, but in a strange place the names at once attract our attention. Mr. Small, in our own town, although he be of great physical stature, does not appeal to us as named out of harmony with himself, and although Mr. Wise be anything but that, it does not occur to us that he is named rather in sarcasm than in truth. But away from home we do not think it too much to expect that Mr. Young shall be youthful in his feelings and behavior, if not in his years; that Mr. Smart shall be mentally bright and personally neat and trim, or that Mr. Short shall be under rather than above the ordinary size, physically, or curt and abrupt in his speech. If we do not find them so, we are liable to a disappointment, which, if only temporary is all the same a disappointment. We forget that away back in the generations, the first Short or Smart or Wise might have been so called as describing or designating him accurately, believing it hardly possible that men who are born with a descriptive name and hearing it constantly as being a part of themselves, do not, unconsciously, try to make the description perfect.

There is no family name, and we may go over the list of our acquaintances if we please, that is not either a mis-description or a no-description of the person bearing it, making it oft times inappropriate if not ridiculous. Few do not know whence and where we of the Anglo-Saxon or Teutonic races obtained our family names. They have been in use but a short time; less than nine hundred years. Even Kings and nobles didn't have any at one time. William, Duke of Normandy, the conqurer of England was only William, and what business had the common people in possessing what their ruler did not? We are descended from those common people, and then they were known as Hwita, the Beekeeper, or Wolsige the Shooter, or Harold the Runner, or in other ways designating their business, their location or some personal characteristic. There was an original John the Smith, called so because he was a worker in metals. The article between the two names being dropped, described him just as accurately. There was an original Brown, also, who must have been noticeably of dark complexion, and an original Baker who made bread. This was all right, but don't we know Smiths and Browns and Bakers who neither toil at anvils, who are blondes and who cannot make bread ? They are not Smiths, nor Browns nor Bakers and it would be ridiculous to call them so, did we but think of it, did not custom sanction it.

Contrast the present with the old way of naming individuals. This way is mere arbitrary or chance nomenclature, and numbers might as well be substituted for the names as has been the case with Kings, Edward I, II, III and IV, or Henry from I up to VIII. That way meant something. If you heard a man's name for the first time you could judge something of that man's history, personal appearance or character. It described and identified him.

There was John the Weaver, and you might be pretty sure that he did what his name imported; or Richard Crookback, and it is certain that his spinal column was more like the letter "S" than like the letter "I." The one was not a doctor or a lawyer, nor the other an Adonis nor an Apollo. And to go further back, is it not pleasant to reflect that when Abraham called to his wife by name, he called as we should now say, "My Princess," for Sarah means just that ? Nor when he wanted the mother of Ishmael, he called her as we should now say "Stranger," for that is what Hagar was. Isaac was "Laughter" in memory of his mother's merriment when she was told that she was to bear a son in her old age; Methusaleh tells us poetically, that he was a man of immense age, his only claim to distinction, for when you repeat his name you say in effect: "Driving away Death;" Moses, by his appellation reveals the fact of his having been saved from drowning by being "drawn out" from the Nile; Alexander is the same as the "Helper of men;" Philip, a "Lover of horses;" Matthew, "A gift." Then in our own vernacular there were Pepin the Short, and Lewis the Fat, of France; William Rufus or the Red, Beauclerc, the Lion-hearted, and Edward Longshanks, of England, and Lorenzo the Magnificent of Florence, not to mention hundreds of others, named so as to be identified. Doubtless humbler people of those times were as distinctly named and how could it be bettered ? Was it not much preferable to the multitude of meaningless John Smiths, William Joneses and Peter Robinsons that fill up the directory of these days ?

That it is so, seems to be indicated by the popular apprehension of the times, for a dissatisfaction as to this later and arbitrary style of naming, is shown to us constantly, making manifest besides that it is a natural attribute of man to give his fellows some distinctive and marked appellation.

Children are never satisfied with the plain John Ryan, Henry Stokes or Richard Roe, that belong to their playmates, and are sure to give to them some name characteristic of some incident, quality or locality. If in no other way their own names are applied in a way to describe them. William, liquid and musical, descends into the sturdy and roystering or contemptuous "Bill," or falls into the familiar and affectionate "Will" or effeminate "Willie." Thomas becomes the impetuous and rollicking "Tom;" Robert, the lively and impatient "Bob," and Samuel, the quick-spoken, urgent "Sam." If some peculiarity marks them, you will be certain to hear it. The play-ground or the school-room will resound with such terms as "Stub," or "Snaky," or "Bricktop," or "Sawed-off," and if you can see the lads to whom they apply, you will at once confess the accuracy of the application.

Even the vicious classes, and they are almost always ignorant, thus coming very near to nature, want some more striking description given of their depraved associates than the ordinary names they bear, and so we have "Red Leary," "Three-fingered Jack," "Mottled Sam," "Sandy Ralph," and hundreds of others. These are called their "aliases;" but it is a mistake. These names describe and identify them; their real names are their "aliases," for they disguise them.

An amusing illustration of this natural tendency is the giving of titles so prevalent in this country. Mr. Snicker, or Stone, or Long mean nothing; but "Judge" Snicker, "Colonel" Stone and "Major" Long are a species of identification, whether deserved or not, that seldom make a miss.

In a still higher range of life the same natural tendency is observable. No man ever rose above his fellow men in any capacity, if he had qualities that drew others to him, who was not named outside of and beyond the name with which he was born, as though there was an inferential acknowledgement that that did not cover the whole case—a sort of a surname being affixed to it as was given to Scipio in old Roman times, making him Scipio Africanus, thus alluding to a conspicuous point in his career, and distinguishing him from the large family of Scipios. We have, in this way, to name only a few out of thousands, "The Little Corporal" following Napoleon to his emperor's throne and beyond it; "Old Pam" designating a famous prime minister of England; "Mad Anthony" of the revolutionary war; "Old Tippecanoe," who won the presidency in 1840; "The Millboy of the Slashes," Tennessee's favorite son; "Scripture Dick," or "The Sage of Binghamton;" "The Little Giant," for the Illinois senator, and "Honest Old Abe," for the war president. And these names, sometimes given in derision, we find clinging to the persons on whom they have been bestowed long after those persons have changed their own names from mere meaningless abstractions into synonyms of high and noble qualities.

What we call christian or given names arose originally in just such a manner as I have been describing, and it seems a little curious that terms so at first used with strong meaning should at length become the merest handles, giving out not a glimmer of sense to those who use them; or, if they do, in many cases vastly changed from the original. The number of these names is smaller than one would suppose. In the English-speaking races there are only fifty-three, and any one of them can be used without an appearance of singularity, for their meaning has long been buried to the ordinary comprehension. Of these fifty-three, we get twenty-five from the Hebrew language, nineteen are derived from the dialects of Western Europe; five are from the Greek, and four from the Latin. Let me name the twelve that are used more than any of the others—Charles, Edward, Francis, George, Henry, James, John, Richard, Robert, Samuel, Thomas and William. Take any one of these, and ask the first person you meet, why Robert? or why Henry? and not as well any other combination of letters, and ten to one you will be answered that they are simply men's names, nothing more; something by which a man may be known and distinguished from his fellow men. An abstract term to mark the individual of a certain genus or family. In one sense, this answer would be right, for that is the use to which the word is put. Yet, as I have said, each of these names has a distinct meaning. Who has not seen a "Henry" who was poor in spirit and purse, and yet when you speak his name, you say, "rich lord." There have been those who have borne the name of Charles, who were cowardly and mean, yet the name signifies "manly" or "noble-spirited," and how many Johns are there who hardly merit the generous appellation given them of "The grace of the Lord?" The converse of these illustrations may make clearer how inharmonious is the misapplication of names. When we see a Grace who embodies in herself a beauty of form and movement that captivates our senses, or a Frank who is outspoken and free, hiding nothing, or a Lily who is delicate and frail, yet charming in her simplicity, are we not prepared to say with some enthusiasm that there is something in a name after all?

So far as family or surnames are concerned, we are helpless, and we must get along with them the best we can. If they are pleasant to the sound, or have an easily apprehended meaning, we may so live as to be worthy of them; if they are rough to the ear, or convey an impression that is unpleasant to the senses, we may still so live that our personality will make those who know us forget the term by which we are called, or fashion it into a meaning full of honor. In the way of christian or given names, parents are the responsible persons, and they have it in their power to load their children down with names that will be a drag upon them all their lives, or to furnish them with such as will be a help to them, creating from the first a pleasant impression, and one that will give some idea of them. In more senses than one is it true that "a good name is better than great riches."

